# Anxiety and Depression Among Patients with Diabetes in Saudi Arabia and Egypt

**DOI:** 10.3390/healthcare12212159

**Published:** 2024-10-30

**Authors:** Madiha Rabie Mahmoud, Ahmed Aljadani, Ammar A. Razzak Mahmood, Reem Falah Alshammari, Mona M. Shahien, Somia Ibrahim, Ashraf Abdel Khalik, Fahaad S. Alenazi, Fayez Alreshidi, Fatma Mohammad Nasr, Hend Faleh Alreshidi, Amal Daher Alshammari, Marwa H. Abdallah, Hemat El-Sayed El-Horany, Kamaleldin B. Said, Abdulrahman M. Saleh

**Affiliations:** 1 Department of Pharmacology, College of Medicine, University of Ha’il, Ha’il 2440, Saudi Arabia; fs.alenazi@uoh.edu.sa; 2 Department of Pharmacology, Theodor Bilharz Research Institute (TBRI), Ministry of Higher Education and Scientific Research, Giza 12411, Egypt; 3 Department of Internal Medicine, College of Medicine, University of Ha’il, Ha’il 2440, Saudi Arabia; 4 Department of Pharmaceutical Chemistry, College of Pharmacy, University of Baghdad, Bab Al-Mouadam, Baghdad 10001, Iraq; amar.mahmoud@copharm.uobaghdad.edu.iq; 5 Department of Family and Community Medicine, College of Medicine, University of Ha’il, Ha’il 2440, Saudi Arabia; refalshammari@uoh.edu.sa (R.F.A.); fs.alreshidi@uoh.edu.sa (F.A.); hendfal@hotmail.com (H.F.A.); amal.alshammari@uoh.edu.sa (A.D.A.); 6 Department of Pediatrics, College of Medicine, University of Ha’il, Ha’il 2440, Saudi Arabia; m.shahin@uoh.edu.sa (M.M.S.); somaiabashir@yahoo.com (S.I.); 7 Department of Intensive Care Unit, TBRI, Ministry of Higher Education and Scientific Research, Giza 12411, Egypt; dr.ashraf.a@hotmail.com (A.A.K.); fatma_elwakeel@live.com (F.M.N.); 8 Department of Pharmaceutics, Faculty of Pharmacy, University of Ha’il, Ha’il 81442, Saudi Arabia; 9 Department of Biochemistry, College of Medicine, University of Ha’il, Ha’il 2440, Saudi Arabia; h.elhorany@uoh.edu.sa; 10 Medical Biochemistry Department, Faculty of Medicine, Tanta University, Tanta 31527, Egypt; 11 Department of Pathology and Microbiology, College of Medicine, University of Ha’il, Ha’il 55476, Saudi Arabia; kbs.mohamed@uoh.edu.sa; 12 Genomics, Bioinformatics and Systems Biology, Carleton University, 1125 Colonel-By Drive, Ottawa, ON K1S 5B6, Canada; 13 Department of Pharmaceutical Chemistry, Faculty of Pharmacy, Cairo University, Kasr El-Aini Street, Cairo 11562, Egypt; abdo.saleh240@azhar.edu.eg; 14 Aweash El-Hagar Family Medicine Center, Epidemiological, Surveillance Unit, Ministry of Health and Population (MOHP), Mansoura 35711, Egypt

**Keywords:** Diabetes Miletus type 2 (T2DM), depression, anxiety, HbA1c, Saudi Arabia, Egypt

## Abstract

Background: Mental stress plagued type II diabetes (T2DM) patients. The psychological and emotional issues related to diabetes and its effects include depression, anxiety, poor diet, and hypoglycemia fear. Aim: Compare the impact of diabetes on depression and anxiety in Egyptian and Saudi diabetics. Methods: The diabetes, gastroenterology, and hepatology sections of University of Ha’il Clinic, KSA, and the Theodor Bilharz Research Institute, Egypt, conducted this retrospective study. Everyone gave informed consent before participating. Interviews with male and female outpatients and inpatients were conducted from June 2021 to December 2022. The self-administered validated Generalized Anxiety Disorder-7 (GAD-7) and the Patient Health Questionnaire-9 (PHQ-9) scale measured sociodemographic characteristics and symptoms of depression and anxiety. Results: In patients with diabetes, the prevalence of depression was higher in KSA [34.8%] than in Egypt [18%], while anxiety was higher in Egypt [40%] than in KSA [29.1%]. Most depressed patients were 31–55 years old (61.2%) from KSA and 97.8% (41–55 years old) from Egypt. Female anxiety was 70.7% in KSA and 51.0% in Egypt, with no significant difference. The duration of diabetes in depressed patients was 5–10 years ([46.9%, Saudis] vs. [57.8%, Egyptians]), while anxious patients (5–10 years [39.0%, Saudis] vs. >20 years [65.0%, Egyptians]) were mainly type-2. Most depressive patients had an HbA1c (59.2%) from 7–10% (Saudis) and 77.8% [>10% Egyptians] compared to anxiety patients (46.3%) and 48.0% [>10% Egyptians]. Depressed and anxious patients from both nations had higher glucose, triglycerides, and cholesterol levels. Saudis and Egyptians with obesity had higher rates of sadness (75.5% vs. 68.9%) and anxiety (82.9% vs. 69.0%). Treatment adherence and serum glucose monitoring were not significantly different from depression in diabetes individuals in both ethnicities. Conclusions: Anxiety was more common among Egyptian patients because of overcrowding, working whole days to fulfill life requirements, and the unavailability of health insurance to all citizens. Meanwhile, in KSA, obesity, unhealthy food, and less exercise reflect the high percentage of depression among patients with diabetes. The detection of depression and anxiety in the context of DM should be critical for the physical health and quality of life of Saudi and Egyptian diabetics. Further investigation is warranted to encompass anxiety and depression within the scope of future research.

## 1. Introduction

According to the International Diabetes Federation, the number of individuals with diabetes is likely to increase from its current estimate of 24 million people aged 20–79 worldwide in 2021 to 33 million by 2030 and 55 million by 2045 [1]. About 8.5% of the world’s population, or 422 million people, had diabetes in 2014, with the majority residing in low- and middle-income nations. Diabetes is also directly responsible for 1.5 million fatalities annually [2]. The World Health Organization (WHO) estimates that 14.4% of Saudi Arabia’s adult population has diabetes mellitus [3]. Meanwhile, the International Diabetes Federation (IDF) places Egypt as having the eighth highest prevalence of DM globally, with an estimated 8,850,400 adult patients with diabetes as of the beginning of the year 2020 [4].

Hazard ratios for mental health disorders range from 0.82 to 3.62, depending on how long it has been since the diagnosis of the mental health issue. This indicates that individuals with mental health disorders are more likely to develop additional medical diseases. Studies indicate a link between mental disorders and a higher risk of type 2 diabetes (T2D). Many factors, such as lifestyle decisions, genetic predispositions, and particularities of the diseases in question, are implicated in this association [5,6,7].

Various diabetes types have various psychosocial impacts. Relationships, jobs, social life, and psychological well-being can all affect quality of life [5]. Type 2 diabetes mellitus is not only a significant chronic disease affecting physical health but also imposes profound psychological burdens on individuals. The relationship between diabetes and mental health is well-established as bi-directional, meaning that the presence of one condition can exacerbate the other. Individuals with diabetes are at an increased risk for developing mental health conditions, including depression and anxiety, both of which have been consistently associated with T2DM across various populations [8].

Diabetes is associated with a higher prevalence of depression than the general population; studies show that people with diabetes are up to three times more likely to suffer depressive symptoms. These depressive symptoms have a strong correlation with the psychological distress related to managing diabetes. In addition, studies showed a 60% greater risk of type 2 diabetes development in individuals with depression [9,10,11].

T2DM shortens life, escalates healthcare costs, and degrades quality of life [12]. Diabetes increases the likelihood of depression and anxiety, two of the most common psychological disorders. Diabetics have twice the risk of anxiety and sadness [10]. Gender, age, education, diabetic complications, and poor blood sugar control are connected to anxiety and sadness in T2DM patients, according to studies [10,13].

In addition, patients with diabetes (type 2) have more anxiety due to difficulty in controlling the illness. Research showed that 14% of people with type 2 diabetes have anxiety disorders and that having anxiety at baseline significantly increases the risk of developing diabetes [14,15]. Depression and anxiety have a major impact on diabetes outcomes. Patients with diabetes with poor mental health are more likely to develop morbidity, high blood glucose, poorer quality of life, and an increased risk of early mortality [13,16,17]. Compared to people without mental health disorders, people with a diagnosis of both diabetes and mental health disorders are more likely to face severe diabetes-related effects, such as cardiovascular, nephropathy, and amputation problems. The psychological burden of managing diabetes, the demands of strict adherence to treatment protocols, and the anxiety related to long-term complications like neuropathy, cardiovascular disease, and vision impairment make effective diabetes management more difficult. Because diabetes and mental health are related, there is a need for integrated care solutions that address both the medical and psychological elements of the illness to improve patient outcomes [18,19].

The Arab community’s views and beliefs about psychiatry are significantly influenced by current and past occurrences, resulting in many difficulties in accepting mental health treatment. The stigma linked to mental health disorders is a major obstacle to receiving medication, particularly for patients with diabetes with emotional distress because of fears of social rejection. Mental illness increases feelings of shame and loneliness, since it is often regarded as a sign of weakness in both Saudi Arabia and Egypt. Attitudes on mental health counseling can be affected by cultural factors, including family relationships. Further medical barriers are worries about privacy, trust in providers, mental health literacy, and practical difficulties [20,21,22].

Saudi and Egyptian people are more likely to acquire T2DM due to physiological, cultural, and socioeconomic factors [23,24]. KSA and Egypt have few comparative studies on T2DM patients’ sadness and anxiety symptoms. We selected Saudi Arabia and Egypt due to their shared cultural background and high DM rate but different economic and healthcare infrastructures. This comparison allows us to explore how these differences affect the mental health outcomes of patients with diabetes. Depression and anxiety in T2DM patients were examined in our study.

## 2. Materials and Methods

### 2.1. Patients and Methods

This prospective study was carried out at the University of Ha’il Clinics, Egypt, by researchers from the Theodor Bilharz Research Institute and the Section of Diabetes and Gastroenterology and Hepatology at University of Ha’il. The study protocol was approved by the Research Ethical Committee (REC) at the University of Ha’il, with research number (H-20221-106) and dated 5 April 2021. Before taking part, everyone gave their informed consent. Male and female patients who were seen in the outpatient clinic over the course of 1.5 years (June 2021–December 2022) were interviewed to compile the data. In our study, we utilized the Generalized Anxiety Disorder-7 (GAD-7) and the Patient Health Questionnaire-9 (PHQ-9) to assess anxiety and depressive symptoms, respectively. The GAD-7 is a self-report measure designed to evaluate the severity of generalized anxiety, consisting of seven items rated on a 0 to 3 scale, with total scores ranging from 0 to 21. A cutoff score of five or higher in the Arabic version indicates clinically relevant anxiety, and it demonstrated acceptable internal consistency with a Cronbach’s alpha of 0.763. The PHQ-9 is used to measure depressive symptoms, comprising nine items rated on a similar 0 to 3 scale, with total scores ranging from 0 to 27. A score of five or higher in the Arabic version was used to identify potential cases of depression, with a Cronbach’s alpha of 0.857, indicating good internal consistency. Both the GAD-7 and PHQ-9 are used for screening purposes and are not intended for formal diagnostic use [25,26,27]. In total, 2346 individuals participated, with 846 Saudi citizens and 1500 Egyptian. We used basic descriptive statistics. Predictors of depressive, anxious, and stressful states were identified.

### 2.2. Inclusion Criteria

All individuals exhibited diabetes mellitus type 2, within the age range of 20 to 70 years and beyond, encompassing both male and female cohorts.

### 2.3. Exclusion Criteria

Patients with chronic kidney disease and pregnant women were not included in the study.

### 2.4. Statistical Analysis

Statistical analysis was carried out using the Statistical Package for Social Sciences (SPSS Inc., Chicago, IL, USA, version 25). Frequencies (*n*) and percentages (%) were used to present categorical variables. According to the Pearson chi-square test, a *p*-value of 0.05 was declared statistically significant.

## 3. Results

**Figure 1:**
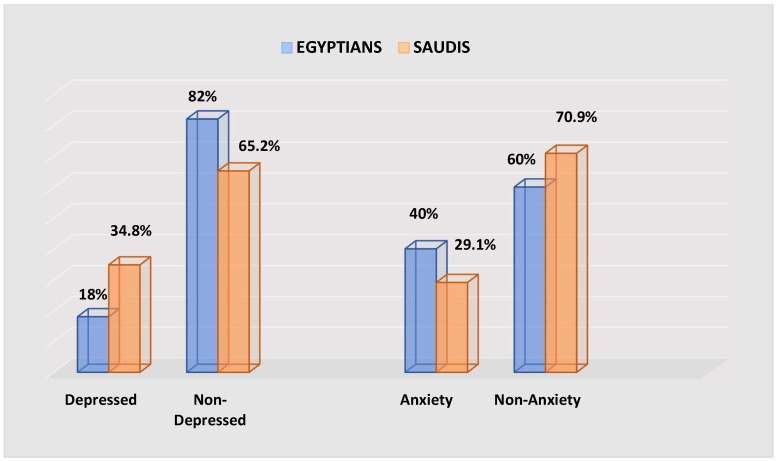
Depression and anxiety rates among Saudi and Egyptian patients with diabetes.

The total number of participants was 2346, with 846 Saudis and 1500 Egyptians. Table 1 displays the demographic features and depression levels of diabetes patients from Saudi Arabia and Egypt. Saudi Arabia had a higher number of depressed patients (34.8%) (Figure 1). The majority of depressive patients in KSA were 31–55 years old (30.6%), compared to 97.8% (41–55 years old) in Egypt, with a considerable age disparity between the two nationalities. About 55.1% of females in KSA and 68.9% of males in Egypt exhibit depressive symptoms. However, there was no significant difference between males and females in KSA. Furthermore, there was no significant difference in education level between depressed patients in KSA, but the difference was significant among depressed Egyptians (*p*-value = 0.011). Obese patients were more likely to be depressed than non-obese patients (Saudis 75.5% vs. Egyptians 68.9%). In terms of smoking, no statistically significant difference was seen between depressed patients in KSA (*p*-value = 0.706) and Egyptians (*p*-value = 0.983). Eating nutritious meals for diabetes and exercising, on the other hand, demonstrated a significant difference in KSA (*p*-value = 0.049 and 0.044) vs. Egyptians (*p*-value = 0.013 and 0.018).

**Figure 2:**
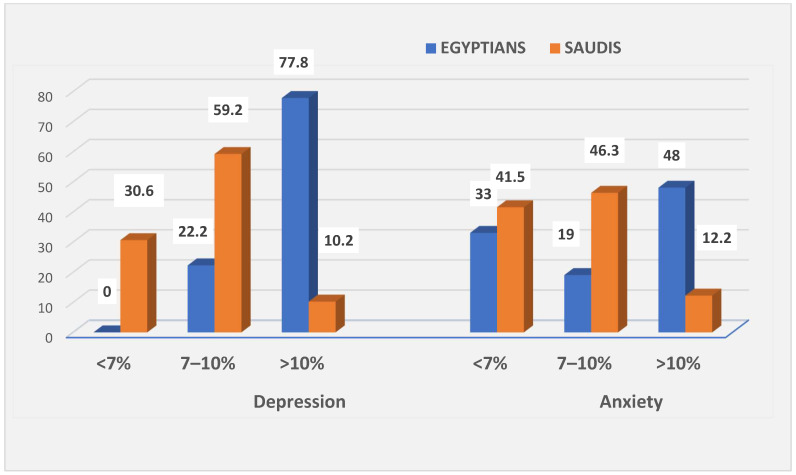
Association between HbA1c and depression or anxiety among Saudi and Egyptian patients with diabetes (%).

Table 2 shows how clinical characteristics affect depression in Egyptian and Saudi diabetics. Most Saudi and Egyptian diabetics had type 2 diabetes, and depressed people had it for fewer than 5–10 years. Depressed individuals in Saudi Arabia had a cumulative HbA1c of 7–10% (59.2%), while Egyptians had >10% (77.8%) (Figure 2).

Among patients with diabetes in both nationalities, there is no significant difference between depressed or non-depressed patients either in their adherence to treatment (*p*-value = 0.638 vs. 0.447), or regularity in measuring serum glucose (*p*-value = 0.229 vs. 0.437). Diabetes and its consequences (on their works) were considerably influenced in depressed patients either in Saudis (*p* = 0.017) or Egyptians (*p* = 0.011), but not influenced in patients without depression.

Table 3 shows how laboratory testing affects depression in Egyptian and Saudi diabetics. Fasting blood sugar, postprandial blood sugar, and hemoglobin were significantly different in depressed patients compared to non-depressed Saudis and Egyptians (*p*-value = 0.018 vs. 0.009). Depressed and non-depressed diabetic Saudis (*p* = 0.495) and Egyptians (*p* = 0.662) had similar thyroid hormone serum levels. Cholesterol, triglycerides, LDL, and HDL were dramatically altered in depressed and non-depressed diabetic Saudis and Egyptians.

Table 4 indicates demographics and anxiety among Saudi and Egyptian diabetics. Egypt had 40% more anxious diabetics (Figure 1). Most anxiety patients in KSA were 61 years old or older (43.9%), compared to 49.0% (41–55 years old) in Egypt. The age gap was significant. About 70.7% and 51.0% of KSA and Egyptian women had anxiety, but no significant difference was found between males and females in Egypt. Anxious Saudis and Egyptians with a medium education differed significantly (Saudis *p*-value = 0.000, Egyptian *p*-value = 0.010). Saudis and Egyptians with obesity have higher anxiety rates (82.9% vs. 69.0%). Smoking did not differ between anxious Saudi patients (*p* = 0.521) and Egyptians (*p* = 0.376). However, KSA and Egyptians differed in a diabetes-friendly diet (*p*-value = 0.041 vs. 0.040) and activity (*p*-value = 0.013 vs. 0.041).

Table 5 shows how clinical characteristics affect anxiety in Egyptian and Saudi diabetics. Most patients with diabetes (Saudis or Egyptians) have type 2 diabetes, and 39.0% had it for less than 5–10 years in KSA. Meanwhile, in Egypt, 65.0% had for more than 20 years. Most anxious patients (46.3%) had an HbA1c of 7–10% (Saudis) and (48.0%) > 10% (Egyptians). Among patients with diabetes of both nationalities, namely Saudis and Egyptians, no significant difference between anxious or non-anxious patients, either in their adherence to treatment (*p*-value = 0.378 vs. 0.828) or regularity in measuring serum glucose (*p*-value = 0.164 vs. *p*-value = 0.889), but patients’ information about diabetes and its medications was significantly different (*p*-value = 0.002 vs. *p*-value= 0.050). Diabetes and its consequences (on their work) were considerably influenced in anxious patients, but not in patients without anxiety, either for Saudis (*p* = 0.017) or Egyptians (*p* = 0.011).

Table 6 shows how laboratory tests affect anxiety in Saudi and Egyptian diabetics. Anxious patients had significantly different fasting, postprandial, and hemoglobin blood sugars (*p*-value = 0.016 vs. 0.049), postprandial (*p*-value = 0.042 vs. 0.011), and hemoglobin (*p*-value = 0.009 vs. 0.027). Thyroid hormone serum levels were not substantially different between anxious and non-anxious diabetic Saudis (*p*-value = 0.921) or Egyptians (*p*-value = 0.480). Cholesterol, triglycerides, LDL, and HDL were dramatically altered in anxious and non-anxious diabetic Saudis and Egyptians, as shown in the table.

## 4. Discussion

This study looked at depression and anxiety in type 2 patients with diabetes from Saudi Arabia and Egypt. The prevalence of depressive symptoms in Saudi patients with T2DM was 34.8%, which is similar to reports from other countries, such as Jordan [28] and Palestine [29], and another study conducted in Saudi Arabia [30]. In comparison to previously published research, our conclusion is virtually as low as the study in Pakistan, which found 49.2% depression in patients with diabetes [31] and in Palestine [32]. According to the findings of this study, the prevalence of depression among Egyptian patients (18.0%) is lower than the prevalence of depression among Saudi patients. These findings are lower than those of the research conducted in Jordan, Palestine, and Pakistan [28,32,33] but greater than those of studies conducted in Malaysia and Qatar [34,35]. The use of different scales to screen for depressed symptoms in patients and the settings utilized in this research could be the causes for the varying prevalence rates of depression in the studies.

While the exact cause of depression is unclear, it is thought to be linked to imbalances in monoamine neurotransmitters, like serotonin (5-HT), dopamine, and norepinephrine [32], all of which have been linked to an increased risk of depression in people with type 2 diabetes. Counterregulatory chemicals, such as catecholamine, glucocorticoids, growth hormones, and glucagon, are thought to be activated in response to emotional stress, preventing insulin from doing its job and instead raising glucose and blood sugar levels [36].

Anxiety is prevalent among individuals with diabetes, in addition to depression, and numerous studies have examined this correlation. In our investigation, anxiety disorders were present in 29.1% of Saudi patients and 40% of Egyptian patients. We suggest that anxiety was more common among Egyptians because of overcrowding, difficulty in traffic, and people being busier in fulfilling life requirements, so they were working all day. Also, health insurance was not available to all citizens, so they were afraid of illness. When he had a chronic disease, he suffered from anxiety. Meanwhile, in KSA, the pace of life is characterized by calm in all dealings, and all citizens are covered by health insurance in the case of illness. So, they do not have excessive tension or anxiety from fear of the lack of health care.

A recent study in Egypt found that significant relationships were discovered between the likelihood of anxiety and various study variables, including gender, peripheral painful neuropathy, retinopathy, the existence of diabetes complications, coma or spasm, history of hypertension, depression, and the number of problems [37].

Our results are in agreement with other results showing that anxiety was less in KSA because patients do not have to worry about treatment costs [38]. The study of Baum et al. (2013) supports our results. They said that education may indirectly affect stress and anxiety through financial support for health and wellbeing, whereas health insurance may directly affect it. Higher education was associated with higher employment rates and better health benefits, such as health insurance, paid leave, and retirement [39]. A prior study related socioeconomic status to anxiety and mood disorders [40].

Anxiety was observed in 50.7%, 43.6%, 38.3%, and 35.3% of patients with DM, according to studies conducted in Pakistan [33], Saudi Arabia [3,30], and Qatar [35], which reported comparable findings to those in Egyptian patients. In Qatar, the prevalence of depression, anxiety, and stress symptoms in DM patients was more than in healthy subjects by 2 fold [35]. In Pakistan, painful peripheral diabetic neuropathy leads to the onset of depression [41], due to neurochemical imbalance [42]. In Qassim (KSA), anxiety was more common among patients who had poor social support but was less common among retired people and those with a DM duration >10 years [30].

On the contrary, a study conducted in Malaysia revealed that anxiety symptoms are present in only 9% of patients with diabetes [34]. Consistent with the findings reported in the Saudi study, other research conducted in 15 countries [43], and in Baltimore, United States [44], found a negligible prevalence of anxiety among type 2 diabetes patients, at 18.0% and 21.8%, respectively. Diverse assessment instruments and the characteristics of the patients enrolled in these investigations could account for the discrepancy in outcomes [31]. In studies conducted in Malaysia anxiety was tenfold more likely to develop in those with depression [34]. Anxiety could be avoided by improved psychological well-being and conscientiousness, which reduced the incidence of anxiety in half patients. In Malaysian and Chinese patients with diabetes, the likelihood of anxiety was considerably increased by the presence of depression [34,45]. It is known that anxiety and depression are positively correlated in people with chronic illnesses and that patients who suffer from depression are more inclined to develop anxiety symptoms [46].

In this study, the percentage of depression (18%) among Egyptians was less than among Saudis (34.8%). In Egypt, about 60% of participants (mainly males, 68.9%) with medium education level, 33.3% with long duration with DM > 20 years, and 22.2% with an HbA1c from 7 to 10%. These results reflect a low percentage of depression in Egyptian patients with diabetes. Also, most of them are aged 41–55 years old (97.8%), and they can perform exercises (90%) and eat healthy food (51.1%). A similar study in Egypt showed that the prevalence of depression in patients with DM was 21.8% but was more common among younger ages and higher education levels [47]. Similar research was conducted to investigate the relationship between various characteristics and depression in Egyptian diabetes patients. Depression was associated with age, female gender, higher education, and exercise. Depression was linked to age, as previously found [48]. Isolation, medical concerns, and limitations make older individuals more susceptible to psychiatric disorders [49]. More than one-third of diabetics in Egypt suffered from anxiety and depression, which may be due to poor clinical outcomes. In addition, age, the duration of diabetes, symptoms of neuropathy, retinopathy, peripheral painful neuropathy, the presence of DM complications, coma or spasm, history of hypertension, and anxiety were all significantly associated with depression [37].

An analysis of this study indicated that, in Saudi diabetes patients, depression was associated with age (61.2% between 31–55 years old, equally from males and females), with a higher education level (40.8%), with a long duration of DM > 20 years, and with an HbA1c from 7 to 10% (59.2%), also obesity (75.5%), a small percentage of healthy eating (32.7%), and a low percentage of patients who were doing exercise (26.5%). These results reflect the high percentage of depression among patients with diabetes. In the western region of KSA, age, sex, the presence of comorbidities, period since T2DM diagnosis, and serum hemoglobin A1c level were significant predictors of psychological distress. The only protective factors were being older and adhering to diabetes management [3]. In Qassim, Saudi Arabia, depression was more common in patients who had received moderate or low social support but less common in those with diabetes > ten years [30]. Diabetes patients are more likely to develop psychological symptoms due to longer diabetes duration, which significantly elevates the risk of complications and medical costs [38].

In contrast to our findings, several studies show that higher education reduces the risk of depression [50]. Meanwhile, other studies revealed that educated people seek medical care more often and are diagnosed with depression and chronic disease [51]. Higher quality of life related to physical health or related to social relationships both reduce the likelihood of depression. Spirituality and religiosity may be protective factors against depression, especially in older adults, according to research [52].

Our data also suggest that Saudi women are much more depressed than men. A recent study found 19.7% of women had depressed symptoms compared to 13.9% of men [53]. The relationship between depression and female hormones like estrogen is contradictory, with research showing both positive and negative associations [54]. As in prior investigations, the female sex predicted depression and anxiety in Egyptian patients but not in Saudi patients. Much research has connected female predominance to a lack of social support and bad life outcomes [55]. Women may be more emotional and extroverted than men due to their social function [48].

Depression and anxiety were more common in type II diabetes in our study. A longitudinal study of the biomarkers of inflammation and depressed symptoms in type 1 and type 2 diabetics supports this [56]. Reduced indicators of inflammation were longitudinally related to reduced depressed symptoms in T2DM patients [57]. No similar relationships were seen in T1DM patients, suggesting that the risk factors and pathomechanisms linking inflammation and depression may vary by diabetes type [56]. In contrast to other studies [57], our data show that patients with diabetes with <10 years duration of diabetes are more prone to have depression than those with >10 years. Like earlier studies, age was linked to anxiety and depression in Saudi and Egyptian patients [48]. Age-related difficulties, like loneliness, sickness, and limitations, make older persons more susceptible to psychological troubles [49].

Different research found inconsistent links between metabolic syndrome and anxiety and depression [11]. Some studies link metabolic syndrome to depression [58]. Others found no correlation [59]. Unstandardized symptom criteria may explain these contradictory results in all such investigations. Elevated systolic BP, fasting BG, and fasting TG were linked to anxiety and sadness [32]. From our findings, we can suggest that obesity and metabolic syndrome components, such as fasting blood sugar, triglycerides, and low HDL, are linked to psychological illnesses. Obesity, a key component of metabolic syndrome, has been linked to depression in diabetics. Depression and metabolic syndrome are related in a lot of manners, especially regarding obesity [59]. In our study, obesity was linked to depression and anxiety in the diabetics of both nations. Recent studies indicated that type 2 diabetes sadness was associated with increased BMI [60].

We found a link between physical inactivity and sadness and anxiety among Saudi and Egyptian patients, most of whom do not exercise. Exercise boosts b-endorphins and brain neurotransmitters, which protect against depression and psychiatric disorders [61]. Khuwaja et al. [62] and Hong et al. [63] observed that physical exercise inversely correlated with anxiety and depression in various populations.

Our study found that sad Saudi diabetics comply less with their diets than Egyptians. Among both nationalities’ diabetics, anxiety was linked to decreased dietary restriction. Other investigations have shown that sad diabetics do not self-care every day [64]. Our findings support previous research that revealed depressed diabetics experience physical limits and a poor quality of life [65], including adhering to food restrictions and exercising [64]. Depressive symptoms predict poor self-care, especially diet and exercise adherence, according to Hapunda et al. [66].

In line with a study that found greater depression and anxiety were associated with poorer behavioral diabetes management and glycemic control among Mexican Americans [67], our study found higher HbA1C in both nationality groups. These data imply that depression and anxiety negatively impact diabetes behavioral management and glucose control. Diabetes, anxiety, and depression are among the top 10 causes of disability-adjusted life years (DALYs) worldwide, according to a study [68].

Our investigation identified anemia in depressive and anxious patients of both nationalities, similar to a Pakistani study [69]. One theory linking depression with anemia is that decreasing muscle power and frailty might lower a patient’s quality of life and lead to depressive symptoms [70].

## 5. Conclusions

Anxiety and depression are prevalent among patients with diabetes in both Egypt and Saudi Arabia. Anxiety was more common among diabetic Egyptian patients because of overcrowding, working the whole day to fulfill life requirements, and the unavailability of health insurance to all citizens. Also, two-thirds of them (anxious patients with diabetes) have a DM duration of >20 years, with half of them having an HbA1c (>10%) and low hemoglobin levels, and about 90% have hyperthyroidism. Meanwhile, in KSA, obesity, unhealthy food, and less exercise reflect the high percentage of depression among patients with diabetes. Also, about only one-third of them (depressed patients with diabetes) adhere to treatment. Two-thirds have a low hemoglobin level, and more than 90% have a bad lipid profile. The diagnosis of depression and anxiety in the context of DM should be critical for the psychiatric health and quality of life of patients with diabetes in Egypt and Saudi Arabia. Further investigation is warranted to encompass melancholy and anxiety within the scope of future research. Awareness programs about DM and psychiatric symptoms should be held in KSA and Egypt due to their great importance.

## Figures and Tables

**Table 1:** Demographics and depression in Egyptian and Saudi diabetics [2346 participants, 846 Saudis, 1500 Egyptians].

Characteristics Total No. 2346	Saudi Patients *n* = 846			Egyptian Patients *n* = 1500		
	Depressed 294 (34.8%)	Non-Depressed 552 (65.2%)	*p*-Value	Depressed 270 (18.0%)	Non-Depressed 1230 (82.0%)	*p*-Value
**Age (years)**						
18–30	18 (6.1)	132 (23.9)	0.030 *	0 (0.0)	186 (15.1)	0.014 *
31–40	90 (30.6)	96 (17.4)		0 (0.0)	402 (32.7)	
41–55	90 (30.6)	144 (26.1)		264 (97.8)	162 (13.2)	
56–60	24 (8.2)	102 (18.5)		6 (2.2)	306 (24.9)	
61 and above	72 (24.5)	78 (14.1)		0 (0.0)	174 (14.1)	
**Gender**						
Male	132 (44.9)	210 (38.0)	0.430	186 (68.9)	636 (51.7)	0.036 *
Female	162 (55.1)	342 (62.0)		84 (31.1)	594 (48.3)	
**Level of education**						
Medium	84 (28.6)	78 (14.1)	0.134	162 (60.0)	312 (25.3)	0.011 *
Secondary	90 (30.6)	204 (37.0)		24 (8.9)	510 (41.5)	
University and above	120 (40.8)	270 (48.9)		84 (31.1)	408 (33.2)	
**Obesity**						
Yes	222 (75.5)	318 (57.6)	0.035 *	93 (68.9)	648 (52.7)	0.009 *
No	72 (24.5)	234 (42.4)		42 (31.1)	582 (47.3)	
**Healthy Foods for DM**						
Yes	96 (32.7)	150 (27.2)	0.049 *	138 (51.1)	312 (25.4)	0.013 *
No	198 (67.3)	402 (72.8)		96 (48.9)	918 (74.6)	
**Doing Exercises**						
Yes	78 (26.6)	276 (50.0)	0.044 *	27 (10.0)	204 (16.6)	0.018 *
No	216 (73.4)	276 (50.0)		243 (90.0)	1026 (83.4)	
**Smoking**						
Yes	150 (51.0)	300 (54.3)	0.706	905 (33.3)	408 (33.2)	0.983
No	144 (49.0)	252 (45.7)		180 (66.7)	822 (66.8)	

* Significant difference at *p*-value ≤ 0.05.

**Table 2:** Association between the clinical characteristics and depression among Egyptian and Saudi Arabia patients with diabetes [Total *n* = 2346 participants, *n* = 846 (Saudis), *n* = 1500 (Egyptians)].

Characteristics Total No. 2346	Saudi Patients *n* = 846			Egyptian Patients *n* = 1500		
	Depressed *n* = 294	Non-Depressed *n* = 552	*p*-Value	Depressed *n* = 270	Non-Depressed *n* = 1230	*p*-Value
**Type of Diabetes**						
Type 1	24 (8.2)	162 (29.3)	0.038 *	18 (6.7)	192 (15.6)	0.046 *
Type 2	270 (91.8)	390 (60.7)		252 (93.3)	1038 (84.4)	
**Duration of DM**						
<5–10 years	138 (46.9)	366 (66.3)	0.047 *	154 (57.8)	426 (34.6)	0.020 *
15–20 years	72 (24.5)	84 (15.2)		24 (8.9)	180 (14.6)	
>20 years	84 (28.6)	102 (18.5)		90 (33.3)	624 (50.8)	
**HbA1c**						
<7	90 (30.6)	372 (67.4)	0.011 *	0 (0.0)	390 (31.7)	0.008 *
7–10	174 (59.2)	174 (31.5)		60 (22.2)	750 (61.0)	
>10	30 (10.2)	6 (1.1)		210 (77.8)	90 (7.3)	
**Adherence to treatment**						
Yes	102 (34.7)	162 (29.3)	0.638	150 (55.6)	708 (57.6)	0.447
No	192 (65.3)	390 (70.7)		120 (44.4)	522 (42.4)	
**Measuring serum Glucose regularly**						
Yes	168 (57.1)	330 (59.8)	0.229	150 (55.6)	750 (61.0)	0.437
No	126 (42.9)	222 (40.2)		120 (44.4)	480 (39.0)	
**Information about DM and its medications**						
Yes	138 (46.9)	396 (71.7)	0.032 *	210 (77.8)	888 (72.2)	0.017 *
No	156 (53.1)	156 (28.3)		60 (22.2)	342 (27.8)	
**Effect of DM or its complication on your work**						
Yes	258 (87.8)	360 (65.2)	0.014 *	246 (91.1)	750 (61.0)	0.012 *
No	36 (12.2)	192 (34.8)		24 (8.9)	480 (39.0)	

* Significant difference at *p*-value ≤ 0.05.

**Table 3:** Laboratory testing and depression in Egyptian and Saudi diabetes patients [2346 participants; 846 Saudis, 1500 Egyptians].

Characteristics Total No. 2346	Saudi Patients *n* = 846			Egyptian Patients *n* = 1500		
	Depressed *n* = 294	Non-Depressed *n* = 552	*p*-Value	Depressed *n* = 270	Non-Depressed *n* = 1230	*p*-Value
**Fasting Blood Sugar**						
Normal	12 (4.1)	42 (7.6)	0.018 *	0 (0.0)	522 (42.4)	0.009 *
High	264 (89.8)	366 (66.3)		240 (88.9)	606 (49.3)	
Do not Know	18 (6.1)	144 (26.1)		30 (11.1)	102 (8.3)	
**Postprandial Blood Sugar (after 2 Hrs.)**						
Normal	12 (4.0)	48 (8.6)	0.019 *	0 (0.0)	186 (15.1)	0.003 *
High	258 (87.8)	366 (66.2)		252 (93.3)	924 (75.1)	
Do not Know	24 (8.2)	138 (25.0)		18 (6.7)	120 (9.8)	
**Hemoglobin**						
Normal	72 (24.5)	42 (7.6)	0.016 *	60 (22.2)	690 (56.1)	0.007 *
Low	192 (65.3)	462 (83.7)		180 (66.7)	388 (31.5)	
Do not Know	30 (10.2)	48 (8.7)		30 (11.1)	336 (27.3)	
**Cholesterol level**						
Normal	18 (6.1)	60 (10.9)	0.008 *	42 (15.5)	480 (39.0)	0.042 *
High	270 (91.9)	330 (59.8)		210 (77.8)	630 (51.2)	
Do not Know	6 (2.0)	162 (29.3)		18 (6.7)	120 (9.8)	
**Triglyceride level**						
Normal	18 (6.1)	84 (15.2)	0.006 *	18 (6.7)	252 (42.4)	0.008 *
High	252 (85.7)	318 (57.6)		216 (80.0)	708 (57.6)	
Do not Know	24 (8.2)	150 (27.2)		36 (13.3)	0 (0.0)	
**LDL**						
Normal	18 (6.1)	96 (17.4)	0.050 *	42 (15.5)	540 (43.9)	0.039 *
High	232 (77.6)	288 (52.2)		204 (75.6)	600 (48.8)	
Do not Know	48 (16.3)	168 (30.4)		24 (8.9)	90 (7.3)	
**HDL**						
Normal	18 (6.1)	102 (18.5)	0.036 *	36 (13.3)	492 (40.0)	0.020 *
Low	210 (71.4)	216 (39.1)		204 (75.6)	618 (50.2)	
Do not Know	66 (22.4)	234 (42.4)		30 (11.1)	120 (9.8)	
**Thyroid Hormones**						
Normal	54 (18.4)	120 (21.7)	0.495 *	0 (0.0)	10 (2.4)	0.662
High	204 (69.4)	396 (71.8)		264 (97.8)	388 (94.6)	
Low	18 (6.1)	24 (4.3)		0 (0.0)	4 (1.0)	
Do not Know	18 (6.1)	12 (2.2)		6 (2.2)	8 (2.0)	

* Significant difference at *p*-value ≤ 0.05.

**Table 4:** Demographics and anxiety in Egyptian and Saudi diabetes patients [2346 participants; 846 Saudis, 1500 Egyptians].

Characteristics Total No. 2346	Saudi Patients *n* = 846			Egyptian Patients *n* = 1500		
	Anxiety *n* = 246 (29.1%)	Non-Anxiety *n* = 600 (70.9%)	*p*-Value	Anxiety *n* = 600 (40%)	Non-Anxiety *n* = 900 (60%)	*p*-Value
**Age (years)**						
18–30	24 (9.8)	126 (21.0)	0.037 *	0 (0.0)	126 (14.0)	0.010 *
31–40	36 (14.6)	150 (25.0)		0 (0.0)	402 (44.7)	
41–55	30 (12.2)	204 (34.0)		294 (49.0)	132 (14.7)	
56–60	48 (19.5)	78 (13.0)		138 (23.0)	174 (19.3)	
61 and above	108 (43.9)	42 (7.0)		168 (28.0)	66 (7.3)	
**Gender**						
Male	72 (29.3)	270 (45.0)	0.084	294 (49.0)	528 (58.7)	0.132
Female	174 (70.7)	330 (55.0)		306 (51.0)	372 (41.3)	
**Level of education**						
Medium	120 (48.8)	120 (20.0)	0.000 *	366 (61.0)	108 (12.0)	0.010 *
Secondary	42 (17.1)	174 (29.0)		120 (20.0)	414 (46.0)	
University and above	84 (34.1)	306 (51.0)		114 (19.0)	378 (42.0)	
**Obesity**						
Yes	204 (82.9)	336 (56.0)	0.003 *	414 (69.0)	354 (39.3)	0.002 *
No	42 (17.1)	264 (44.0)		186 (31.0)	546 (60.7)	
**Healthy Foods for DM**						
Yes	72 (29.3)	348 (58.0)	0.041 *	294 (49.0)	204 (22.7)	0.040 *
No	174 (70.7)	252 (42.0)		306 (51.0)	696 (77.3)	
**Doing Exercises**						
Yes	60 (24.4)	306 (51.0)	0.013 *	0 (0.0)	204 (22.7)	0.041 *
No	186 (75.6)	294 (49.0)		600 (100)	696 (77.3)	
**Smoking**						
Yes	90 (36.6)	186 (31.0)	0.521	210 (35.0)	258 (28.7)	0.376
No	156 (63.4)	414 (69.0)		390 (65.0)	642 (71.3)	

* Significant difference at *p*-value ≤ 0.05.

**Table 5:** Clinical features and anxiety in Egyptian and Saudi Arabian diabetes patients [2346 participants; 846 Saudis, 1500 Egyptians].

Characteristics Total No. 2346	Saudi Patients *n* = 826			Egyptian Patients *n* = 1500		
	Anxiety *n* = 246	Non-Anxiety *n* = 600	*p*-Value	Anxiety *n* = 600	Non-Anxiety *n* = 900	*p*-Value
**Type of diabetes**						
Type 1	24 (9.8)	150 (25.0)	0.034 *	72 (12.0)	210 (23.3)	0.050 *
Type 2	222 (90.2)	450 (75.0)		528 (88.0)	690 (76.7)	
**Duration of DM**						
<5–10 years	96 (39.0)	408 (68.0)	0.017 *	174 (29.0)	420 (46.7)	0.000 *
15–20 years	66 (26.8)	90 (15.0)		36 (6.0)	162 (18.0)	
>20 years	84 (34.2)	102 (17.0)		390 (65.0)	318 (35.3)	
**HbA1c**						
<7(%)	102 (41.5)	360 (60.0)	0.005 *	198 (33.0)	192 (21.3)	0.000 *
7–10 (%)	114 (46.3)	234 (39.0)		114 (19.0)	702 (78.0)	
>10 (%)	30 (12.2)	6 (1.0)		288 (48.0)	6 (0.7)	
**Adherence to DM medications**						
Yes	150 (61.0)	438 (73.0)	0.378	396 (66.0)	582 (64.7)	0.828
No	96 (39.0)	162 (27.0)		204 (34.0)	318 (35.3)	
**Measuring serum glucose regularly**						
Yes	126 (51.2)	354 (59.0)	0.164	504 (84.0)	750 (83.3)	0.889
No	120 (48.8)	246 (41.0)		96 (16.0)	150 (16.7)	
**Information about DM and its medications**						
Yes	120 (48.8)	456 (76.0)	0.002 *	360 (60.0)	780 (86.7)	0.050 *
No	126 (51.2)	144 (24.0)		240 (40.0)	120 (13.3)	
**Effect of DM or its complication on your works**						
Yes	216 (87.2)	402 (67.0)	0.017 *	516 (86.0)	420 (46.7)	0.011 *
No	30 (12.2)	198 (33.0)		84 (14.0)	480 (53.3)	

* Significant difference at *p*-value ≤ 0.05.

**Table 6:** Laboratory tests and anxiety in Egyptian and Saudi patients with diabetes [2346 participants; 846 Saudis, 1500 Egyptians].

Characteristics Total No. 2346	Saudi Patients *n* = 846			Egyptian Patients *n* = 1500		
	Anxiety *n* = 246	Non-Anxiety *n* = 600	*p*-Value	Anxiety *n* = 600	Non-Anxiety *n* = 900	*p*-Value
**Fasting Blood Sugar [*n* (%)]**						
Normal level	24 (9.8)	192 (32.0)	0.016 *	186 (31.0)	192 (21.3)	0.049 *
High level	204 (82.9)	384 (64.0)		384 (64.0)	666 (74.0)	
Do not Know	18 (7.3)	24 (4.0)		30 (5.0)	42 (4.7)	
**Postprandial Blood Sugar (after 2 h.)** **[*n* (%)]**						
Normal level	12 (4.9)	408 (68.0)	0.042 *	54 (9.0)	198 (22.0)	0.011 *
High level	216 (87.8)	168 (28.0)		172 (86.0)	660 (73.3)	
Do not Know	18 (7.3)	24 (4.0)		30 (5.0)	42 (4.7)	
**Hemoglobin** **[*n* (%)]**						
Normal level	84 (34.1)	462 (77.0)	0.009 *	306 (46.0)	570 (63.3)	0.027 *
Low level	132 (53.7)	90 (15.0)		294 (49.0)	186 (20.7)	
Do not Know	30 (12.2)	48 (8.0)		30 (5.0)	144 (16.0)	
**Cholesterol level** **[*n* (%)]**						
Normal level	18 (7.3)	60 (10.0)	0.039 *	48 (8.0)	360 (40.0)	0.015 *
High level	210 (85.4)	390 (65.0)		480 (80.0)	366 (40.7)	
Do not Know	18 (7.3)	150 (25.0)		72 (12.0)	174 (19.3)	
**Triglyceride level** **[*n* (%)]**						
Normal level	24 (9.8)	84 (14.0)	0.026 *	60 (10.0)	276 (30.7)	0.022 *
High level	204 (82.9)	366 (61.0)		468 (78.0)	4505 (50.0)	
Do not Know	18 (7.3)	150 (25.0)		72 (12.0)	174 (19.3)	
**LDL [*n* (%)]**						
Normal level	48 (19.5)	252 (42.0)	0.006 *	78 (13.0)	336 (37.3)	0.004 *
High level	180 (73.2)	198 (33.0)		450 (75.0)	390 (43.4)	
Do not Know	18 (7.3)	150 (25.0)		72 (12.0)	174 (19.3)	
**HDL [*n* (%)]**						
Normal level	60 (24.4)	210 (35.0)	0.041 *	244.0)	312 (34.7)	0.002 *
Low level	168 (68.3)	240 (40.0)		504 (84.0)	414 (46.0)	
Do not Know	18 (7.3)	150 (25.0)		72 (12.0)	174 (19.3)	
**Thyroid Hormones** **[*n* (%)]**						
Normal	54 (22.0)	150 (25.0)	0.921	18 (3.0)	42 (4.7)	0.480
High	156 (63.4)	414 (69.0)		558 (93.0)	738 (82.0)	
Low	18 (7.3)	24 (4.0)		12 (2.0)	30 (3.3)	
Do not Know	18 (7.3)	12 (2.0)		12 (2.0)	90 (10.0)	

* Significant difference at *p*-value ≤ 0.05.

## Data Availability

All data is available within the article.
